# Development of an artificial synovial fluid useful for studying *Staphylococcus epidermidis* joint infections

**DOI:** 10.3389/fcimb.2022.948151

**Published:** 2022-07-29

**Authors:** Johanna Stamm, Samira Weißelberg, Anna Both, Antonio Virgilio Failla, Gerhard Nordholt, Henning Büttner, Stefan Linder, Martin Aepfelbacher, Holger Rohde

**Affiliations:** ^1^ Institut für Medizinische Mikrobiologie, Virologie und Hygiene, Hamburg, Germany; ^2^ Microscopy Imaging Facility, University Medical Center Hamburg-Eppendorf, Hamburg, Germany; ^3^ Institute for Clinical Chemistry, Universitätsklinikum Hamburg-Eppendorf, Hamburg, Germany; ^4^ Deutsches Zentrum für Infektionsmedizin, Standort Hamburg-Lübeck-Borstel, Hamburg, Germany

**Keywords:** joint infection, prosthetic joint infection (PJI), *Staphylococcus epidermidis (S. epidermidis)*, biofilm formation, confocal laser scanning electron microscope

## Abstract

*Staphylococcus epidermidis* is a major causative agent of prosthetic joint infections (PJI). The ability to form biofilms supports this highly selective pathogenic potential. *In vitro* studies essentially relying on phenotypic assays and genetic approaches have provided a detailed picture of the molecular events contributing to biofilm assembly. A major limitation in these studies is the use of synthetic growth media, which significantly differs from the environmental conditions *S. epidermidis* encounters during host invasion. Building on evidence showing that growth in serum substantially affects *S. epidermidis* gene expression profiles and phenotypes, the major aim of this study was to develop and characterize a growth medium mimicking synovial fluid, thereby facilitating research addressing specific aspects related to PJI. Using fresh human plasma, a protocol was established allowing for the large-scale production of a medium that by biochemical analysis matches key characteristics of synovial fluid and therefore is referred to as artificial synovial fluid (ASF). By analysis of biofilm-positive, polysaccharide intercellular adhesion (PIA)-producing *S. epidermidis* 1457 and its isogenic, PIA- and biofilm-negative mutant 1457-M10, evidence is provided that the presence of ASF induces cluster formation in *S. epidermidis* 1457 and mutant 1457-M10. Consistent with the aggregative properties, both strains formed multilayered biofilms when analyzed by confocal laser scanning microscopy. In parallel to the phenotypic findings, expression analysis after growth in ASF found upregulation of genes encoding for intercellular adhesins (*icaA*, *aap*, and *embp*) as well as *atlE*, encoding for the major cell wall autolysin being responsible for eDNA release. In contrast, growth in ASF was associated with reduced expression of the master regulator *agr*. Collectively, these results indicate that ASF induces expression profiles that are able to support intercellular adhesion in both PIA-positive and PIA-negative *S. epidermidis*. Given the observation that ASF overall induced biofilm formation in a collection of *S. epidermidis* isolates from PJI, the results strongly support the idea of using growth media mimicking host environments. ASF may play an important role in future studies related to the pathogenesis of *S. epidermidis* PJI.

## Introduction

*Staphylococcus epidermidis* is a leading pathogen isolated from prosthetic joint infections (PJI) (Becker et al., 2020), a serious challenge for the modern healthcare system (Schwarz et al., 2019). The marked increase in joint replacement procedures (Patel et al., 2015), in combination with a constant infection rate of 1%–3% (Lourtet-Hascoet et al., 2016), has led to a steep increase in the number of PJI cases. Considering the significant morbidity and mortality associated with PJI (Izakovicova et al., 2019; Natsuhara et al., 2019), there is an urgent need for novel preventive and therapeutic approaches. Detailed insights into the molecular pathogenesis of *S. epidermidis* PJI are regarded as an essential brick in this strategy. In fact, tremendous progress on the road to a detailed molecular picture of *S. epidermidis* implant infections has been made, unraveling a plethora of mechanisms contributing to successful device colonization.

The ability to assemble multilayered biofilms is key to the opportunistic *S. epidermidis* virulence in PJI (Both et al., 2021). Biofilm formation is essentially promoted by the production of an extracellular matrix, which by embedding bacterial cells functions as a glue that fosters cell aggregation. The matrix consists of polysaccharides [i.e., polysaccharide intercellular adhesin (PIA)], proteins [e.g., accumulation-associated protein (Aap), extracellular matrix-binding protein (Embp), or small basic protein (Sbp)], and extracellular DNA (eDNA, released through the activity of autolysin AtlE). Intriguingly, epidemiological studies have produced partially conflicting results by identifying clinically significant *S. epidermidis* that were unable to form a biofilm *in vitro* (Rohde et al., 2007). The discrepancy can at least in part be explained by the use of convenient growth media (e.g., TSB), which support the formation of PIA- but not protein-dependent biofilms (Christner et al., 2012). The functional relevance of PIA-independent biofilm formation through the production of Embp only became apparent by supplementing TSB with serum or tigecycline, leading to *embp* expression (Christner et al., 2010; Weiser et al., 2016). Clearly, growth in TSB only is a very poor approximation to the *in vivo* environment, and thus, relevant mechanisms related to *S. epidermidis* pathogenesis may have gone unidentified by their use. Particularly for PJI, studies provided evidence that *in vitro* experiments in convenient laboratory media do not reflect bacterial growth *in vivo* under conditions of a human joint cavity (Skovdal et al., 2021; Steixner et al., 2021). Bacteria entering a human joint cavity have to adapt to the unique environment provided by the synovial joint fluid (SF), which lubricates the joint cavity. SF is able to induce the formation of bacterial aggregates (Bidossi et al., 2020) or biofilms adhering to the implant device or tissue within the joint cavity (Barrett and Atkins, 2014; Gbejuade et al., 2015), and thus, SF obviously promotes the expression of pathogenesis-relevant phenotypes. Despite this fact, however, only a few studies have so far been conducted using unmodified human SF (Dastgheyb et al., 2015; Gilbertie et al., 2019). Typically, human SF is diluted before use (Knott et al., 2021; Staats et al., 2021; Steixner et al., 2021), or substituted by animal SF (Gupta et al., 2021; Perez and Patel, 2015; Ibberson et al., 2016; Pestrak et al., 2020; Staats et al., 2021). The obvious limitations of these approaches currently have to be accepted due to the limited availability of human SF (Gilbertie et al., 2019; Steixner et al., 2021). While substitute joint fluid to treat joint disease has already been introduced into modern medicine (Adams et al., 1995; Neustadt et al., 2005; Faust et al., 2018), only a few attempts to use a *bona fide* artificial SF in pathogenesis research have been made (Knott et al., 2021).

Taking into account the importance of using media closely mimicking the *in vivo* environment for studying *S. epidermidis* infection biology, the major aim of this study was to develop and characterize an artificial synovial fluid (ASF). By resembling human joint fluid and being available in larger quantities, ASF may substantially contribute to a more precise identification and characterization of *S. epidermidis* traits relevant to the pathogenesis of PJI.

## Methods

### Bacterial strains

*S. epidermidis* 1457 (Galac et al., 2017) is a PIA-producing, biofilm-positive isolate from a central venous catheter infection. 1457-M10 is a corresponding isogenic, biofilm-negative transposon mutant carrying Tn*917* in *icaA*, interfering with the biosynthesis of PIA (Mack et al., 2000). In addition, 23 previously characterized *S. epidermidis* isolated from PJI were used (Both et al., 2021).

### Growth analysis

Bacteria were grown overnight in TSB at 37°C and shaking (200 rpm). Cultures were diluted in fresh TSB or ASF at a ratio of 1:100, and 200 µl were transferred into wells of a 96-well microtiter plate (Sarstedt, Nümbrecht, Germany). Plates were incubated for 24 h at 37°C in a microplate reader (Agilent, Santa Clara, CA, USA). Absorption at 600 nm was measured every hour as a surrogate for bacterial growth.

### Quantification of biofilm formation

Biofilm formation was quantified using a 96-well microtiter plate assay essentially as described previously (Both et al., 2021).

### Analysis of sessile bacterial cultures by confocal laser scanning microscopy

Bacteria grown on coverslips were mildly washed with PBS and then fixed with 4% paraformaldehyde (PFA) in PBS for 10 min. Unspecific binding sites were blocked using 3% bovine serum albumin (BSA, w/v) in PBS for 1 h. Next, samples were incubated with a 1:500 dilution of α-dsDNA primary antibody (Abcam, Cambridge, UK) for 1 h, washed three times with PBS, and incubated with a 1:250 dilution of an α-mouse IgG coupled to A488 (ThermoFisher, Waltham, MD, USA). For visualization of bacteria, 300nM DAPI (Invitrogen, Carlsbad, CA, USA) was added to the secondary staining solution and both antibodies were applied in PBS supplemented with 1.5% BSA. Coverslips were mounted with MOWIOL anti-fade reagent (Calbiochem, Darmstadt, Germany), and microscopic analysis was carried out using a Leica TCS SP8 confocal laser-scanning instrument.

### Analysis of bacterial cell cluster analysis

For analysis of bacterial cluster size and size distribution, bacteria were grown statically for 18 h at 37°C in TSB and ASF, respectively. Thirty minutes prior to staining, the bacterial sediment was carefully dispersed by vortexing, and 10 µl were transferred to staining buffer (3% BSA in PBS + DAPI 300 nM, Invitrogen) in a µ-Slide 8-Well (ibidi, Gräfelfing, Germany). Samples were analyzed with the confocal laser scanning microscope Leica TCS SP8 equipped with a ×63, NA1.4 oil immersion objective and LAS X SP8 software (Leica Microsystems, Wetzlar, Germany). At least 10 positions per condition were recorded as stacks with a distance of 500 nm and 2,048 × 2,048 pixels. Bacterial detection and segmentation were done using the Imaris software package (Oxford Instruments, Abingdon, UK). Subsequently, cluster analysis was done in MatLab (Version 9.2, The MathWorks Inc., Natick, MA, USA). In brief, the position of each bacterium was calculated by its volume, and the center of mass of the segmented volume was defined. Distances between centers of masses were measured and categorized into clusters with a threshold of ≥5 bacteria/cluster. The MatLab script used is available in **MatLab Supplement S1**.

### Gene expression analysis

For gene expression analysis, bacteria were grown in triplicates in TSB overnight at 37°C under vigorous shaking. Cultures were then diluted in TSB or ASF and grown for 6 and 24 h at 37°C with shaking (180 rpm). Cells were then harvested by centrifugation, washed in PBS, and resuspended in RNAprotect (Qiagen, Hilden, Germany). After incubation at RT, bacterial cells were pelleted. The resulting pellet was mechanically lysed with zirconia beads 3 × 20 s on a tissue homogenizer (Precellys 12, Bertin, Montigny-le-Bretonneux, France), and RNA was extracted with the RNeasy Mini Kit (Qiagen, Hilden, Germany). Bacterial RNA was quantified with a Qubit fluorometer (ThermoFisher Scientific, Waltham, MD, USA) followed by digestion of residual DNA with a DNA-Free Kit (Invitrogen, Carlsbad, CA, USA). A total of 5-µl digested RNA was transcribed using iScript cDNA Synthesis Kit (BioRad, Hercules, CA, USA) according to the manufacturer’s instructions. Quantification of gene expression was carried out using the Light Cycler 480 instrument and applying the TaqMan Fast Advanced Master Mix 2 × (ThermoFisher Scientific, Bremen, Germany). Primers and probes to quantify the expression of *gyrB*, *aap*, *ica*, *atlE*, *agr*, and *embp* are given in **Table 1**.

**Table 1:** Primers and probes used in this study.

Target gene	Primers/probes	Sequence (5′-3′)
*gyrB*	gyrB_fwd	TGGTCTGCGTTCATTTCACCAAGAC
	gyrB_rev	CTTGCCGATGTTGATGGTGCACA
	gyrB_probe	FAM-GGCGGCTGAGCAATATAAACGTAGCCCGC-BHQ-1
*aap*	aap fwd	AACATTAGAGTAGCAAACAATCGTCAAAGTA
	aap rev	AGCCTTGACCAGCTTGTTTCTGTA
	aap probe	FAM-ACAACTGGTGCAGATGGTTGGGGC-BHQ-1
*icaA*	icaA fwd	TGCCTTATTTATTGACAGTCGCTACG
	icaA rev	CGTTGGATATTGCCTCTGTCTGG
	icaA probe	FAM-ATACTGGGTTATCAATGCCGCAGTTGTCA-BHQ-1
*embp*	embp fwd	CACCTGGTGCTGTGCGTAATATAC
	embp rev	GCAGTTCCGTTATTTGTTGGTCCG
	embp probe	FAM-ATGGTCGTTGGACTGTTGAAACTGGGTC-BHQ-1
*atlE*	atlE/R fwd	GATCACGCTGACCCTCACCAAT
	atlE/R rev	GCAACACCACGATTAGCAGACAC
	atlE/R probe	FAM-GCAAGTAGCACCTTGGGGCACAACATC-BHQ-1
*agr*	agr fwd	TGTTGGCAAACTTTCAATAGCACCATG
	agr rev	TCGTGTCGCAGCACTTACAACAACGA
	agr probe	FAM-TCGTGTCGCAGCACTTACAACAACGA-BHQ-1

### Human plasma and clinical chemistry analysis of blood products

Human plasma was obtained from healthy volunteers after giving informed consent. Pools from at least five individuals were used for ASF production. Clinical chemistry analysis of pooled plasma samples was performed according to standard protocols for routine analytics in patient care at the University Medical Center Hamburg-Eppendorf.

## Results

### Development of an artificial synovial fluid

Studies of human joint fluid have indicated for decades that synovial fluid appears to be a dialysate of human plasma. The qualitative composition of protein components, therefore, does not differ between plasma and SF (Decker et al., 1959; Hui et al., 2012; Waldrop et al., 2014), and thus it appeared reasonable to use human plasma as a basis for the intended artificial synovial fluid. Considering reference concentrations from the literature (**Table 2**), a dilution of plasma by half was expected to meet immunoglobulin concentrations, while the remaining protein components would marginally exceed or fall below the target joint fluid values. As synovial fluid electrolytes meet actual plasma concentrations, Jonosteril (Fresenius Kabi, Bad Homburg, Germany), an electrolyte medium developed to match actual human plasma electrolyte concentrations, was employed as the diluent. Intriguingly, using plasma 1:1 (vol/vol) diluted in Jonosteril as a medium, no bacterial growth was detectable. In fact, measuring glucose levels found concentrations of 36 mg/dl, which is clearly below the expected joint fluid glucose concentrations of 60–90 mg/dl. Given that glucose is an important metabolite for staphylococcal growth (Dobinsky et al., 2003; Jager et al., 2005; Agarwal and Jain, 2013; Waldrop et al., 2014; Luo et al., 2020), Jonosteril-diluted plasma was therefore supplemented with glucose to a final concentration of 79 mg/dl.

**Table 2:** Comparison of ASF with human synovial fluid composition.

	Human synovial fluid^a^ ^,^ ^b^	ASF^c^
**Electrolytes**		
- Sodium	138.11 mmol/L	144 mmol/L
- Potassium	5.48 mmol/L	3.5 mmol/L
- Calcium	2.39 mmol/L	1.8 mmol/L
- Chloride	108.41 mmol/L	97.9 mmol/L
- Magnesium	1.47 mg/dl	0.94 mmol/L
**Glucose**	60–95 mg/dl	79 mg/dl
**pH**	7.31–7.64	7.52
**Total protein**	19–28 g/L	28 g/L
**Albumin**	12 mg/ml	16.1 mg/ml
**Albumin**	56%	60.2%
**α_1_-Globulin**	8%	3.9%
**α_2_-Globulin**	7%	8.8%
**β-Globulin**	11%	>16.3%
**γ-Globulin**	18%	<10.8%
**Complement factor C3**	Not available	39.2 mg/dl
**Complement factor C4**	Not available	6.8 mg/dl
**IgA**	approx. ½ plasma concentration	1.11 g/L
**IgG**	approx.½ plasma concentration	3.62 g/L
**IgM**	approx. ½ plasma concentration	0.38 g/L
**IgE**	approx. ½ plasma concentration	21.5 UI
**Uric acid**	3.0–7.0 mg/dl	1.7 mg/dl
**Lactate**	9–16 mg/dl	4.94 mg/dl
**Creatinine**	1.06 mg/dl	0.19 mg/dl

a (Decker et al., 1959; Cummings and Nordby, 1966; Levick, 1981; Madea et al., 2001; Mundt, 2010; Hui et al., 2012; Srettabunjong et al., 2019).

b Synovial fluid was largely sampled postmortem from individuals with no history of joint disease.

c ASF was analyzed after freezing, storage at −80°C, and defreezing.

**Figure 1:**
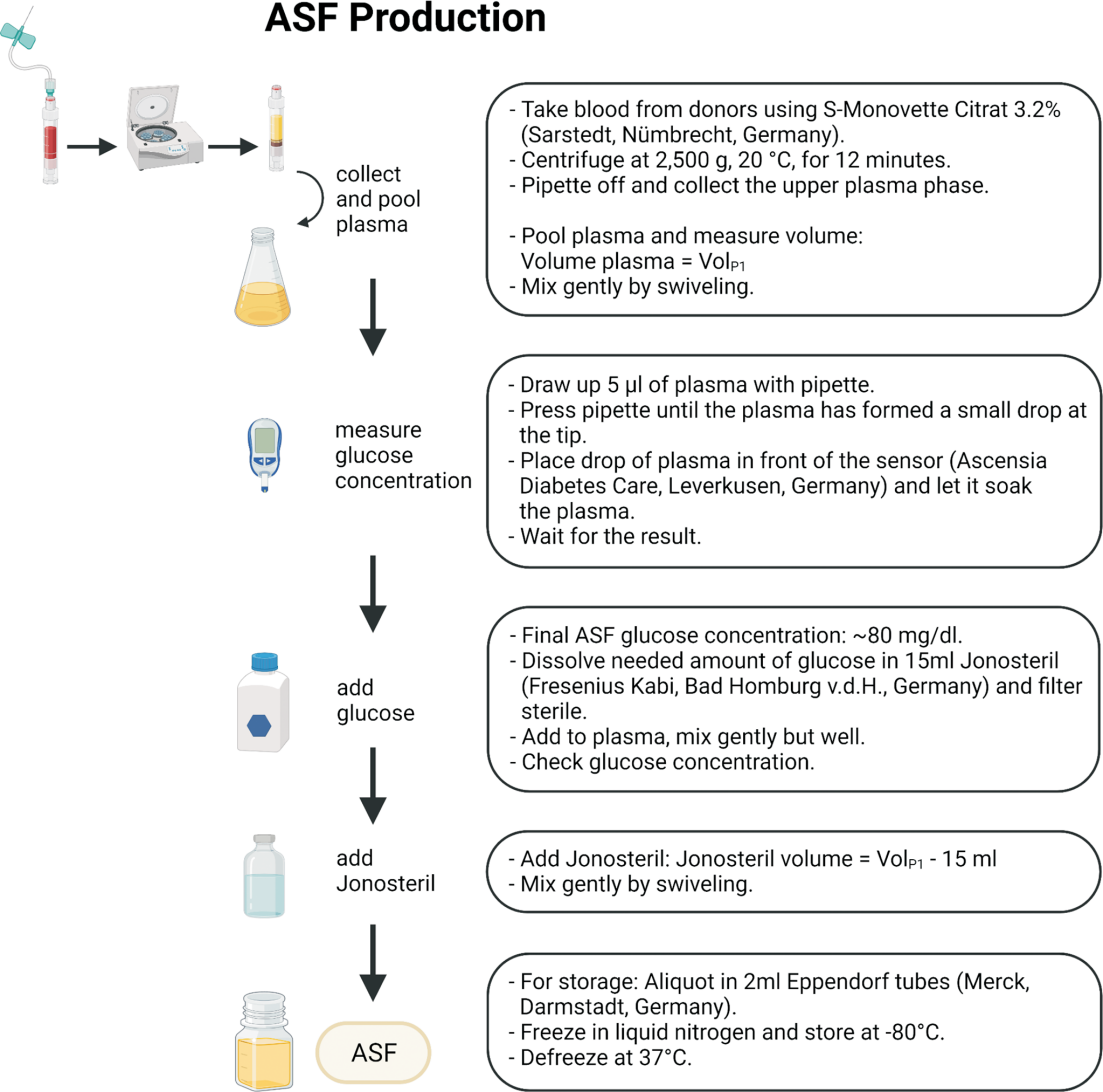
Schematic workflow for ASF production. A more detailed description and necessary consumables can be found in **Supplementary Tables S1–S3**. The figure was created using Biorender.com.

Subsequent biochemical analysis of the glucose-supplemented, Jonosteril-diluted plasma found key component concentrations largely similar to reported concentrations from human synovial fluid (**Table 2**), which is therefore referred to as ASF. **Figure 1** and **Supplementary Table S1** provide a step-by-step protocol describing the production of ASF. Likewise, a protocol for convenient storage of larger ASF volumes is provided, being an important prerequisite to avoiding batch-to-batch variability in larger series of experiments (**Supplementary Table S2**).

### The impact of ASF on growth characteristics, biofilm formation, cell aggregation, and gene expression profiles

**Figure 2:**
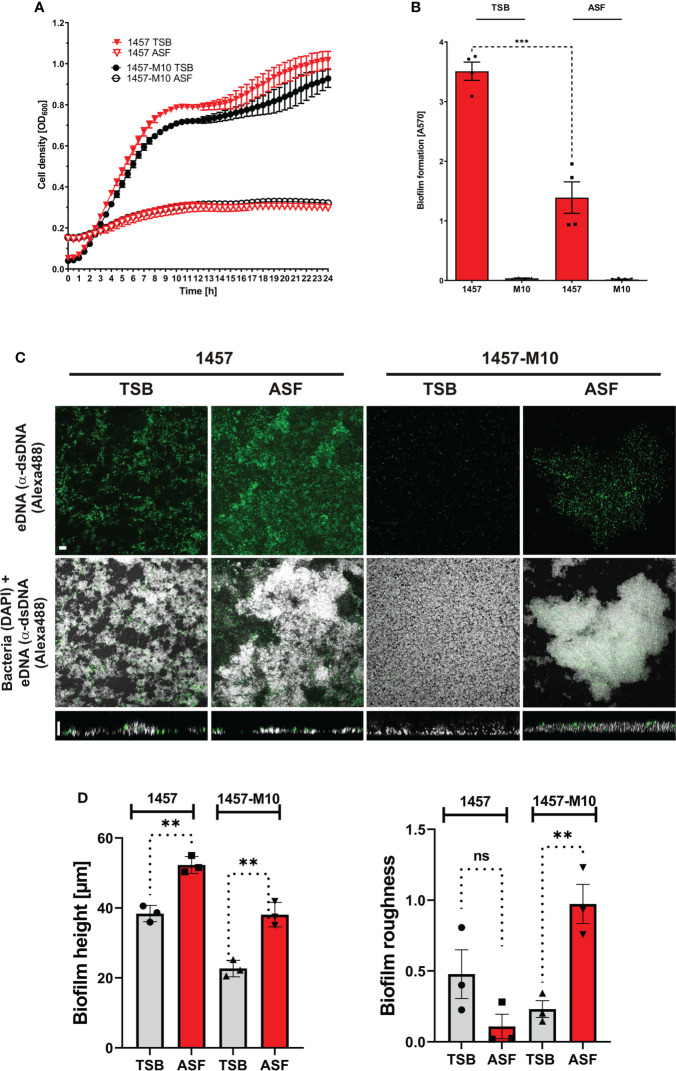
Phenotypic analysis of *S. epidermidis* 1457 and 1457-M10 in TSB and ASF. **(A)** Growth analysis of *S. epidermidis* 1457 and 1457-M10 in TSB and ASF. Absorbance at 600 nm was measured as a surrogate for bacterial growth. Data points represent the mean of two duplicates from two biological replicates. Error bars: standard deviation. **(B)** Analysis of biofilm formation by *S. epidermidis* 1457 and 1457-M10 in TSB and ASF using a microtiter plate assay. After 24 h of growth, the medium was removed, and wells were washed with PBS. After drying, adherent cells were stained using Gentiana violet, and absorbance was read at 570 nm. *S. epidermidis* 1457 formed statistically less biofilm in ASF compared to TSB (unpaired *t*-test; p<= 0.001). Bars represent the mean of two quadruplicates from two biological replicates. Error bars: standard deviation. **(C)** CLSM analysis of *S. epidermidis* 1457 and 1457-M10 after overnight sessile culture in TSB and ASF. Bacteria were grown for 24 h on glass coverslips, and adherent cells were fixed after mild washing. Bacteria were stained using DAPI (300 nM), and eDNA was detected using mouse anti-dsDNA IgG (1:500) and anti-mouse IgG coupled to Alexa488 (1:250). The upper panel shows the maximum sum projection of dsDNA; the middle panel shows the maximum sum projection of merged DAPI and Alex488 channels (scale bar, 9 µm); and the lower panel shows an XZ-view of merged channels (scale bar, 5 µm). **(D)** Quantitative image analysis of *S. epidermidis* 1457 and 1457-M10. Biofilm height (left panel) and surface roughness (right panel) were determined using BiofimQ (Hartmann et al., 2021) and Comstat (Heydorn et al., 2000) software packages, respectively. Bars represent the mean of three biological replicates with at least 10 analyzed pictures. ns, not significant, *p* > 0.05; ^**^ *p* ≤ 0.01; ^***^ *p* ≤ 0.001.

Previously published work has demonstrated that serum or serum components have a significant impact on phenotypes and expression profiles in *S. epidermidis* from prosthetic joint infections (Both et al., 2021). Building on the hypothesis that growth in ASF will impact *S. epidermidis* physiology, key aspects of *S. epidermidis* pathogenicity were comparatively studied in ASF and the reference medium TSB. To this end, we made use of the well-characterized, prototypic polysaccharide intercellular-adhesin (PIA)-producing, biofilm-forming *S. epidermidis* 1457 and isogenic, PIA- and biofilm-negative mutant 1457-M10. Growth analysis over 24 h found that in TSB and ASF, growth followed a sigmoidal function (**Figure 2A**). However, growth in TSB and ASF in both strains differed with respect to the slope of the curve and the maximum cell density. The resulting lower areas under the curves (AUCs) (AUC_1457_TSB: 15.58 [CI, 15.43–15.73]; AUC_1457_ASF: 2.87 [CI, 2.788–2.953]; AUC_M10_TSB: 14.14 [CI, 13.97–14.30]; AUC_M10_ASF: 3.097 [CI, 3.080–3.113]) indicate the overall reduced bacterial growth in ASF. Importantly, no growth differences were identified between S*. epidermidis* 1457 and mutant 1457-M10.

Biofilm formation was analyzed using a 96-well microtiter plate assay, providing integrated information on adherence and biofilm accumulation. In addition, confocal laser scanning microscopy (CLSM) was employed as a tool to obtain detailed insights into the architecture of sessile *S. epidermidis* populations. Using TSB as a growth medium, *S. epidermidis* 1457 forms robust, PIA-dependent biofilms, while PIA-negative mutant 1457-M10 is unable to assemble a multilayered cell architecture (**Figure 2B**). In line with these findings, CLSM analysis of adherent cell populations found biofilm structures being formed by *S. epidermidis* 1457 after 20 h, whereas 1457-M10 did not exhibit structured multicellular growth (**Figure 2C**). Growth in ASF dramatically changed biofilm phenotypes in both assay systems. Strikingly, using the microtitre plate assay, *S. epidermidis* 1457 produced significantly less biofilm in ASF compared to growth in TSB, while there was no detectable difference in 1457-M10 (**Figure 2B**). CLSM analysis of sessile growth showed that 1457 still forms a huge adherent cell architecture (**Figure 2C**). Compared to TSB, these were significantly higher after growth in ASF (mean height of 38.4 vs. 52.3 µm; *p* = 0.002 (unpaired *t*-test); **Figure 2D**). Markedly, in strong contrast to growth in TSB, 1457-M10 also assembled surface adherent aggregates after 20 h of growth (**Figure 2C**). In fact, bioinformatics image analysis showed that the mean biofilm height in ASF (38.09 µm) was significantly (*p* = 0.0032; unpaired *t*-test) higher compared to growth in TSB (mean height of 22.68 µm; **Figure 2D**). In line with this, the surface roughness of 1457-M10 cultures was significantly (*p* = 0.008, unpaired *t*-test) greater compared to growth in TSB, indicating induction of structured and aggregative growth (**Figure 2D**). No difference was identified in surface roughness that became evident in *S. epidermidis* 1457 (*p* = 0.13; unpaired *t*-test). Collectively, these data indicate that growth in ASF, while reducing biofilm growth in PIA-producing *S. epidermidis* 1457, induces cell surface adherent growth in PIA-negative mutant 1457-M10.

**Figure 3:**
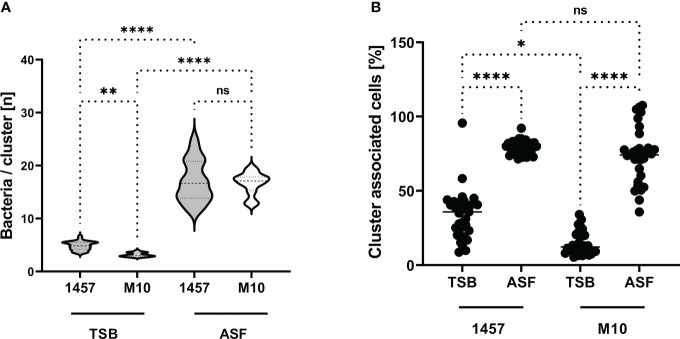
Aggregative cell growth in TSB and ASF. **(A)** Violin plot showing the distribution of cell cluster sizes formed by *S. epidermidis* 1457 and mutant 1457-M10 in TSB and ASF after overnight growth. The size of bacterial cell clusters was determined by bioinformatics analysis of CLSM images. Per strain and growth conditions, at least 30 images from three independent experiments were analyzed. Statistical analysis was done by one-way ANOVA and Sidak’s multiple comparison test (1457 vs. 1457-M10 in TSB: CI of difference: 0.4799–2.926, p = 0.002 1457 vs. 1457-M10 in ASF: CI of difference: −0.8991–1.590, p<= 0.001 1457-M10 TSB vs. 1457-M10 ASF: CI of difference: −14.77 to −12.14, p<=0.001 1457 TSB vs. 1457 ASF: CI of difference: −13.39 to −10.80, p<= 0.001). **(B)** Quantification of *S. epidermidis* cells organized in clusters containing five or more cells. Bioinformatics analysis identified the proportion of bacteria relative to the absolute number of bacterial cells associated with cells to form clusters of ≥5 cells. Each data point indicates the relative proportion (%) of cells meeting this criterion in one image. At least 30 images from three independent experiments were analyzed; the total number of cells ranged from *n* = 10,151 to *n* = 31,094. Results were analyzed using one-way ANOVA with Kruskal–Wallis test for multiple comparisons (adjusted *p*-values: 1457 TSB vs. 1457 ASF, p<= 0.001 1457 TSB vs. 1457-M10 TSB, p= 0.008 1457 ASF vs. 1457-M10 ASF, p= 0.008 1457-M10 TSB vs. 1457-M10 ASF, p<= 0.001 ns, not significant. *p ≤ 0.05; **p ≤ 0.01; ***p ≤ 0.001.

Biofilm formation essentially depends on intercellular adhesion, which phenotypically becomes apparent by the formation of cell aggregates. To test the idea that ASF induces PIA-independent cell aggregative properties and subsequent biofilm formation, experiments were set out to quantify cell cluster size in *S. epidermidis* 1457 and 1457-M10 after growth in TSB and ASF (**Figure 3A**). As expected, after 24 h of growth in TSB, PIA-producing *S. epidermidis* 1457 forms cell aggregates (mean bacteria/cluster (*n*) = 4.9), being slightly but significantly (*p* = 0.0032) larger compared to PIA-negative 1457-M10 (mean bacteria/cluster (*n*) = 3.2). Growth in ASF, however, resulted in the formation of huge, macroscopically visible cell aggregates in 1457 and 1457-M10, and the mean number of cells per cluster was not statistically different between both strains (1457: mean bacteria/cluster: 16.9; 1457-M10 (mean bacteria/cluster: 16.6; *p* = 0.891; **Figure 3A**). Induction of cell aggregation became also evident when the percentage of cell clusters (aggregates ≥5 cells) organized by bacteria was analyzed. In TSB cultures of *S. epidermidis* 1457, significantly (*p* = 0.0106) more bacteria (mean cluster-organized cells, 33.99%) were organized in clusters compared to 1457-M10 (mean cluster-organized cells, 14.61%) (**Figure 3B**). In ASF cultures, however, bacteria of both strains were predominantly organized in clusters (mean cluster-organized cells 1457: 79.9%; 1457-M10: 73.9%; **Figure 3B**), and there was no apparent statistically significant difference (*p* = 0.788). Collectively, these data provide evidence that growth in ASF leads to PIA-independent cell cluster formation, which links growth in ASF with the acquired ability of 1457-M10 to establish a multilayered biofilm architecture as detected by CLSM.

**Figure 4:**
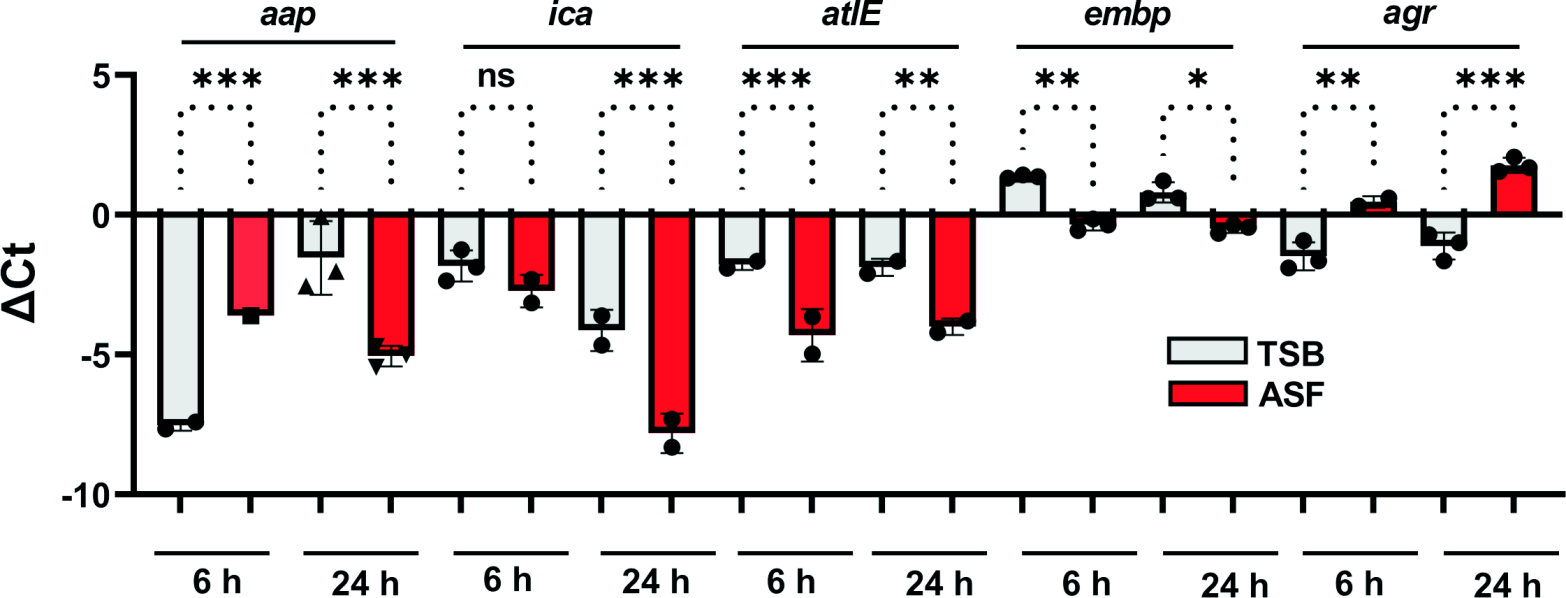
Expression analysis of biofilm-related genes. Relative expression of biofilm-associated genes in *S. epidermidis* 1457 in TSB and ASF. Columns indicate mean ΔCt values obtained from three experiments using independent RNA preparations. The error bars indicate the standard deviation. Differences were analyzed using one-way ANOVA with Holm–Sidak’s multiple comparisons test. *gyrB* served as a reference housekeeping gene. ns, not significant, *p* > 0.05; ^*^ *p* ≤ 0.05; ^**^ *p* ≤ 0.01; ^***^ *p* ≤ 0.001.

Building on data demonstrating a significant impact of growth in the presence of serum on *S. epidermidis* transcriptomes (Both et al., 2021) and expression of *embp* (Christner et al., 2010), qPCR experiments were set out to characterize the expression of genes related to biofilm formation and regulation (**Figure 4**). Expression analysis of biofilm-related *icaA*, *embp*, *aap*, and *atlE* found significantly increased expression in ASF after 24 h of growth. While for *embp* and *atlE*, upregulation in ASF became evident also after 6 h of growth, no significant difference in expression levels was detected for *icaA*. As an exception, *aap* exhibited significantly lower expression levels in ASF after 6 h. Intriguingly, compared to TSB, a master regulator of staphylococcal virulence, *agr* was significantly downregulated after growth in ASF after 6 and 24 h of growth (**Figure 4**).

### Impact of ASF on biofilm formation and cell cluster formation in *S. epidermidis* PJI isolates

**Figure 5:**
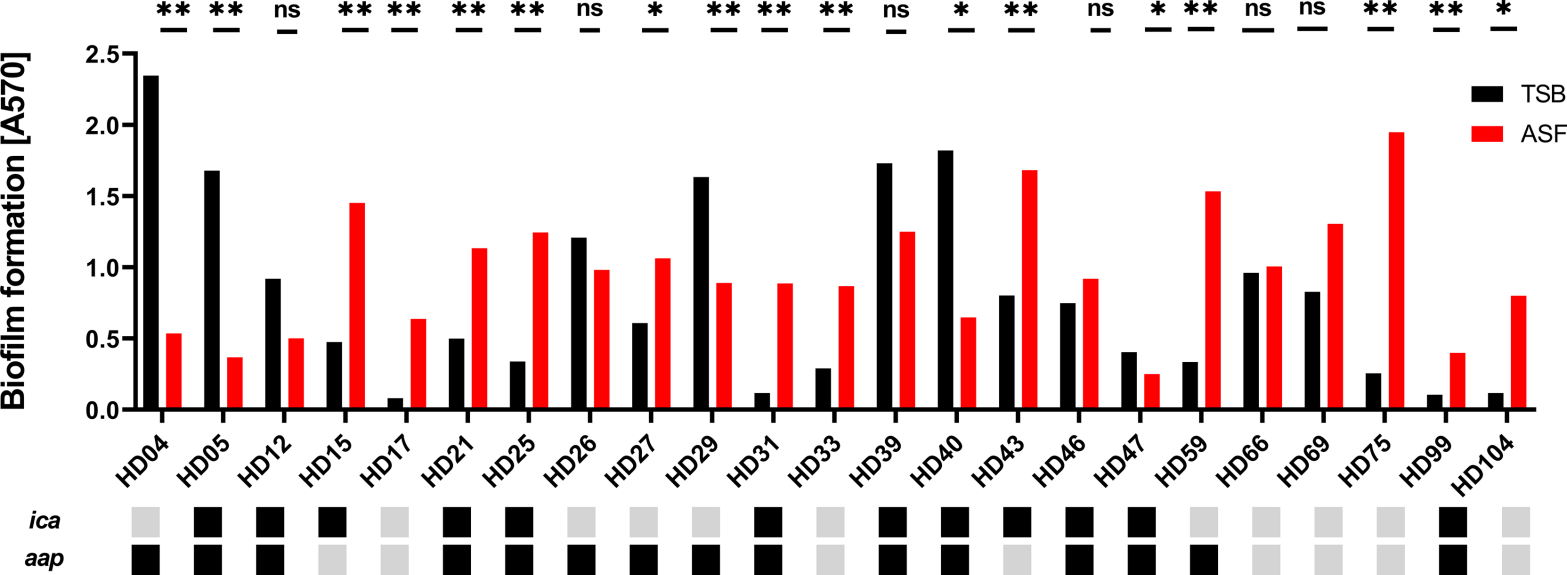
Phenotypic analysis of *S. epidermidis* from PJI. Biofilm phenotypes of 23 *S. epidermidis* isolates from proven PJI were determined using TSB or ASF as a growth medium. Bars represent the mean of eight data points obtained from two experiments. Error bars: standard deviation. The presence and absence of biofilm-related genes *ica* and *aap* are indicated by black and grey boxes, respectively. Pairwise comparison of biofilm quantities produced in ASF and TSB, respectively, was done using the Mann–Whitney *U* test with Holm–Sidak’s multiple comparisons test. ns, not significant, *p* > 0.05; ^*^ *p* ≤ 0.05; ^**^ *p* ≤ 0.01.

The ability to form biofilms is subject to significant, isolate-dependent variation (Rohde et al., 2007). In order to obtain more robust insights into the effects of ASF on biofilm formation, a contemporary collection of *n* = 23 *S. epidermidis* isolated from PJI (Both et al., 2021) were tested for biofilm-forming ability in ASF in comparison to TSB. Overall, quantitative biofilm formation was significantly different between both media tested and between isolates (*p* < 0.0001; two-way ANOVA) (**Figure 5**). In fact, significant differences (Mann–Whitney *U* test with Holms–Sidak’s method for multiple comparisons) were identified in 17/23 isolates tested. In 12 of those 17 isolates (70.6%), growth in ASF increased, and in 5/17 (29.4%) exposure to ASF resulted in weaker biofilm formation Biofilm reduction predominantly occurred in *S. epidermidis* isolates forming strong biofilms in TSB (mean A570/TSB = 1.69; [range, 0.9–2.34]), while increased biofilm formation was identified in isolates exhibiting weak or moderate biofilm formation (mean A570/TSB = 0.4 [range, 0.08–0.83]).

## Discussion

Studies on *S. epidermidis* phenotypes related to infections associated with indwelling medical devices have almost exclusively been carried out using synthetic media, e.g., TSB (Mack et al., 2001). The use of commercially available media significantly supports the reproducibility of experimental results and, thus, is of major importance in an effort to provide an experimental reference supporting the comparability of data from independent research groups. In fact, work based on those artificial media has provided phenotypic and genetic traits associated with invasive *S. epidermidis* lifestyles (Otto, 2018). Intriguingly, by testing genetically defined mutants in comparison with isogenic wild-type strains in animal models of device infections, evidence was provided that mechanisms identified under artificial growth conditions are indeed functionally relevant *in vivo* (Nguyen et al., 2020). However, owing to the obvious limitations of using synthetic media, studies on *S. epidermidis* pathogenicity were carried out using media that resemble the within-host situation, including the presence of host immune effector cells in whole blood assays (Fredheim et al., 2011; Al-Ishaq et al., 2015) or nutrient-poor microenvironments in the nose (Krismer et al., 2014). Support for the need to expand experimental systems and include more life-like growth models comes from recent observations that the presence of host serum has a major impact on *S. epidermidis* expression profiles, including genes related to biofilm formation (Christner et al., 2010; Both et al., 2021). Moreover, it became evident that synovial joint fluid (SF) induces aggregative staphylococcal growth *in vivo* and *in vitro* (Perez and Patel, 2015; Bidossi et al., 2020; Staats et al., 2021), underscoring the need for alternative, more life-like culture systems. To reflect the *in vivo* situation for studying *S. epidermidis* PJI pathogenesis, we, therefore, established a simple protocol for the preparation of an ASF.

Biochemical analysis of ASF found good overall agreement with published data of SF composition from healthy individuals. There are, however, evident discrepancies. While sodium and chloride concentrations agree with reported values, higher magnesium and lower potassium and calcium concentration in ASF was noted. This can partially be explained considering that SF references derive from studies relying on postmortem sampled SF (Madea et al., 2001; Srettabunjong et al., 2019), and forensic studies showed that postmortem degradation processes also influence potassium levels with increasing concentrations relatively linear over time after death (Tumram et al., 2011). Thus, it appears reasonable to assume that the physiological SF potassium concentration is below the literature values and maybe even ranges within the serum concentration range of 3.5 up to 5.0 mmol/L (Tumram et al., 2011; Srettabunjong et al., 2019).

Apart from electrolyte concentrations, SF protein content might be of essential importance for the growth behavior of *S. epidermidis*. A study determining total protein concentrations in SF sampled postmortem and from healthy volunteers showed a range of mean total protein concentrations from 1.3 to 2.8 g/dl with a number adjusted mean of 1.49 g/dl. Higher total protein concentrations were, however, predominantly found in postmortem samples, prompting the conclusion that a total protein concentration of 1.3 g/dl should be considered the healthy reference (Weinberger and Simkin, 1989). Thus, ASF’s total protein concentration of 2.8 g/dl is probably too high. It needs to be stressed, though, that evidence on reference SF values for healthy joints is scarce, probably due to the lack of respective SF samples, and in fact, more recent publications selectively focus on the analysis of SF in joint disease (Iadarola et al., 2016; Peffers et al., 2019). This might also explain why, for complement factors, only data on pathological SF are available (Ochi et al., 1980).

In addition to the total protein concentration, the composition of the SF proteome might differ from plasma proteins due to the presence of specific factors selectively produced within the joint. ASF will therefore show too low concentrations for proteins subject to tissue-specific expression patterns (Bennike et al., 2014), e.g., hyaluronic acid or lubricin (Anderson and Anderson, 2002; Hui et al., 2012; McNary et al., 2012), which are apparently absent in ASF. Thus, ASF must be considered an approximation for a joint fluid model, especially as long as more valid information on physiological SF composition is lacking. Given the evidence from *S. aureus* showing that hyaluronic acid contributes to the aggregate formation (Knott et al., 2021; Staats et al., 2021), the obvious insufficient conformity of ASF with native SF composition might have important experimental implications that must be considered when relating experimental results to the *in vivo* situation. Another obvious limitation of ASF is that it rather reflects healthy than pathological SF, e.g., found in noninfective joint diseases like osteoarthritis (OA) or rheumatoid arthritis (RA). In addition, invasive *S. epidermidis* disease itself will also induce changes in SF composition, potentially affecting bacterial physiology. Despite those limitations, though, it appears unrealistic at present to accommodate for such disease-associated SF changes given actual knowledge gaps in relation to the specific composition of SF in defined joint conditions [e.g., during surgery, after joint implantation, in defined joint diseases (Yazar et al., 2005; Hu et al., 2006; Balakrishnan et al., 2014; Lee et al., 2020; Timur et al., 2021)].

In order to evaluate the usefulness of ASF to study *S. epidermidis* pathogenicity, the impact of ASF on phenotypic traits and gene expression patterns was analyzed using the well-characterized PIA-producing *S. epidermidis* reference strain 1457 and a corresponding, biofilm- and PIA-negative mutant 1457-M10. Here, evidence was obtained that ASF induced cell cluster formation and biofilm formation, resembling microscopic findings from independent studies investigating multicellular architectures in *S. epidermidis* from SF (Perez and Patel, 2015). Intriguingly, aggregative behavior was also identified in PIA-negative mutant 1457-M10, underscoring the importance of PIA-independent mechanisms becoming functionally active during invasive lifestyles (Rohde et al., 2005; Rohde et al., 2007; Skovdal et al., 2021). The induction of *embp* and *aap* expression, as well as increased eDNA release, pinpoint some of the potentially involved mechanisms (Rohde et al., 2007; Christner et al., 2010; Christner et al., 2012). A recent study showed that in the presence of serum factors, *S. epidermidis* cell aggregation may occur independently from Embp and PIA (Skovdal et al., 2021). Evidence of enhanced biofilm formation in genetically independent, clinical *S. epidermidis* isolates in the presence of ASF supports the hypothesis that host factors are relevant to inducing pathogenicity-associated *S. epidermidis* phenotypes *in vitro*. Intriguingly, similar to the observation of reduced biofilm formation in PIA producing *S. epidermidis* 1457, wild-type PJI *S. epidermidis* strains forming strong biofilms in TSB were impaired in accumulative growth in ASF. In contrast, ASF-induced biofilm formation was predominantly observed in *S. epidermidis* wild-type isolates, exhibiting weak biofilm formation in TSB. The regulator *sarA* has previously been shown to negatively control biofilm formation in PIA-negative *S. epidermidis* while supporting protein-dependent biofilm formation in PIA-negative *S. epidermidis* backgrounds (Christner et al., 2012). The involvement of *sarA* in PJI isolates under investigation here is unclear, nevertheless, it appears plausible to assume that *S. epidermidis* possess specific regulatory networks to predominantly promote biofilm formation in the presence of host factors. Thus, more life-like growth models in combination with recent insights into genetic backgrounds of invasive *S. epidermidis* populations as revealed by high-resolution *S. epidermidis* population genomics (Lee et al., 2018; Both et al., 2021; Du et al., 2021; Mansson et al., 2021), have a great potential to link genetic findings with functional phenotypic outputs (e.g., biofilm formation, aggregation).

Master regulator of staphylococcal virulence Agr (Le and Otto, 2015) was significantly downregulated during growth in ASF. Given the evidence that in *S. epidermidis* differential expression of *agr* is a common trait in isolates from invasive disease (Vuong et al., 2004; Olson et al., 2014; Harris et al., 2017), this finding supports the idea that at least, ASF partially resembles the *in vivo* situation. Thus, ASF could serve as an important complementary tool to allow for genetic identification of novel factors contributing to multicellular behavior during the invasion and PJI pathogenesis, e.g., using transposon mutagenesis approaches, transcriptomics, or metabolomics (Mack et al., 2001; Both et al., 2021; Du et al., 2021). In the future, ASF might also be a basis for more advanced models of PJI, e.g., by adding specific macromolecular components (e.g., hyaluronic acid), host immune cells (e.g., macrophages), or by applying shear stress.

Taken together, ASF produced according to the presented protocol expands the toolbox to study *S. epidermidis* PJI pathogenesis, and it might also be used in studies analyzing independent microorganisms relevant to PJI. Thus, ASF has the potential to significantly improve our understanding of the pathogenesis of opportunistic, biofilm-forming *S. epidermidis*. It is reasonable to assume that this knowledge will eventually also translate into better preventive, diagnostic and therapeutic strategies to combat disabling PJI in the future.

## Data availability statement

The raw data supporting the conclusions of this article will be made available by the authors, without undue reservation.

## Ethics statement

The studies involving human participants were reviewed and approved by Ethikkommission der Ärztekammer Hamburg. The patients/participants provided their written informed consent to participate in this study.

## Author contributions

JS: performed experiments, analyzed data, wrote the manuscript. SW: performed experiments, analyzed data, wrote the manuscript. AB: Designed the study, analyzed data, edited the manuscript. AF: analyzed data, provided resources, edited the manuscript. GN: provided resources, edited the manuscript. HB: performed experiments, analyzed data, edited the manuscript. SL: designed experiments, analyzed data, edited the manuscript. MA: provided resources, edited the manuscript. HR: designed the study, analyzed data, wrote the manuscript.

## Acknowledgments

This study was funded by the Damp Foundation (2013-2019, given to HR) and the German Center for Infection Research (DZIF; TI 07.003, given to JS). The authors are grateful for the expert technical assistance of Paul Haffke. We thank all the volunteers who provided plasma and supported the study.

## Conflict of interest

The authors declare that the research was conducted in the absence of any commercial or financial relationships that could be construed as a potential conflict of interest.

## Publisher’s note

All claims expressed in this article are solely those of the authors and do not necessarily represent those of their affiliated organizations, or those of the publisher, the editors and the reviewers. Any product that may be evaluated in this article, or claim that may be made by its manufacturer, is not guaranteed or endorsed by the publisher.
