# Supplementary material for: Development of an artificial synovial fluid useful for studying Staphylococcus epidermidis joint infections
Source: Front Cell Infect Microbiol. 2022 Jul 29;12:948151. doi: 10.3389/fcimb.2022.948151 (PMC9374174; doi:10.3389/fcimb.2022.948151)
Supplement: Supplementary file 1 [file DataSheet_1.docx]

**Table S1: ASF production protocol**

| **Step** |  | **Critical advice** |
| --- | --- | --- |
| **1** | Collect blood: Draw human blood using S-Monovette Citrat 3,2% ( Sarstedt, Nümbrecht, Germany). | Fasting blood draw is required. |
| **2** | Centrifuge the tubes at 2500g for 12 minutes, 20°C. |  |
| **3** | The upper plasma phase is pipetted off carefully using a 10ml serological pipette and collected. Pool plasma using a coverable vessel to avoid contamination. | Leave out the remaining 2mm of the plasma phase, as solid blood components should not be aspired.  Handle plasma and ASF in a sterile environment and wear gloves! |
| **4** | Measure the collected plasma volume. |  |
| **5** | Prepare the same volume of Jonosteril (Fresenius Kabi, Bad Homburg vor der Höhe, Germany) as plasma volume. The final ASF volume consists of 50% plasma and 50% Jonosteril. |  |
| **6** | Measure the glucose level: Insert the sensor into the Contour Next (Ascensia Diabetes Care, Leverkusen, Germany) blood glucose meter and make sure the device is ready. Draw up 5µl of the pooled plasma using a 10µl pipette. Press the pipette until the plasma has formed a drop at the tip. Place the drop in front of the sensor so that the test stripe soaks the plasma and wait for the result. | The sensor only soaks the medium when placed at the tip of the sensor; do not place the drop on top. |
| **7** | Calculate the required amount of glucose that has to be added considering the final ASF volume (see step 5) and the target glucose concentration of 80mg/dl. |  |
| **8** | Dissolve the required amount of glucose in 15ml Jonosteril, vortex 1min and filter sterile using a syringe with sterile filter on top. Then add the solution to plasma and mix well but gently. |  |
| **9** | Check the glucose level with blood glucose meter as described in step 6. Repeat step 7 and 8 if necessary. |  |
| **10** | Add the rest of the prepared volume Jonsteril and mix well but gently. |  |

**Table S2: ASF storage protocol**

| **Step** |  | **Critical advice** |
| --- | --- | --- |
| **1** | Aliquot 1.5ml portions of ASF in 2ml reaction tubes (Eppendorf, Hamburg, Germany). |  |
| **2** | Completely submerge the tubes in liquid nitrogen until ASF is frozen, then store at -80°C. | ASF needs to be frozen quickly and afterwards transported on ice! |
| **3** | Defreeze ASF at 37°C static until ASF is entirely liquid. |  |
| **4** | After defrosting, store ASF at 4°C if necessary. | Do not freeze ASF more than once. |

**Table S3: Equipment and consumables**

| **Item** | **Material as used in this study** |
| --- | --- |

| Centrifuge (Heraeus Multifuge 1 S-R) | Thermo Fisher, Waltham, Massachusetts, United States |
| --- | --- |
| Blood glucose meter (Contour XT) and Contour Next sensor | Ascensia Diabetes Care, Leverkusen, Germany |
| Disinfectant (Cutasept) | Bode Chemie, Hamburg, Germany |
| D-glucose | Merck, Darmstadt, Germany |
| Reaction tubes (Eppendorf safe-lock 2ml) | Eppendorf, Hamburg, Germany |
| Tubes (Falcon 15ml) | Thermo Fisher, Waltham, Massachusetts, United States |
| Freezer (Herafreeze Basic -80°C) | Thermo Fisher, Waltham, Massachusetts, United States |
| Gloves | Remesco, Vienna, Austria |
| Incubator (Certomat BS-1) | Sartorius Stedim Biotech, Göttingen, Germany |
| Jonosteril | Fresenius Kabi, Bad Homburg vor der Höhe, Germany |
| Membrane Filter Unit (Millex-GP Millipore Express PES) | Merck, Darmstadt, Germany |
| Nitrogen, liquid |  |
| Pipettor (Pipetboy) | Integra, Princeton, New Jersey, United States |
| Pipette, 10µl | Eppendorf SE, Hamburg, Germany |
| Pipette tip 10µl | Sarstedt, Nümbrecht, Germany |
| Serological pipette 10ml | Greiner Bio-One, Kremsmünster, Austria |
| S-Monovette Citrat 3,2%, 8,2 ml; order nr. 01.1606.001 | Sarstedt, Nümbrecht, Germany |
| Syringe, 25ml | Becton Dickinson, Franklin Lakes, New Jersey, United States |
| Blood sampling system (Venofix Safety syringe) | Braun, Melsungen, Germany |
| Vortex | Bender & Hobein, Munich, Germany |
