# Supplementary material for: Development of an artificial synovial fluid useful for studying Staphylococcus epidermidis joint infections
Source: Front Cell Infect Microbiol. 2022 Jul 29;12:948151. doi: 10.3389/fcimb.2022.948151 (PMC9374174; doi:10.3389/fcimb.2022.948151)
Supplement: Supplementary file 2 [file DataSheet_2.docx]

% the goal of this script is to determine cluster information starting from

% the results extracted from IMARIS 3D segmentation

clear all

clast_par=1.3;

filename = 'Test_Analysis_1.xls';

%The only two input necessary are the name of the file to be analized i.e file name and parameter that control how far away two

% segmented volumes should be. clast_par < 1 means the distance between two segmented volume should be inferior to the sum of their mean radius

% clast_par =1 the distance between two segmented volumes should be equal to the sum of their mean radius

% clast_par >1 the distance between two segmented volumes should be bigger than the sum of their mean radius

% the data to be quantified is stored in an excel multipage file

%%%%%%%%%%%%%%%%%%%%%%%%%%%%%%%%% segmented volumes radius determination

read_volume=xlsread(filename,32);% opens page where the volumes are listed and extracts the volume information for each cluster

siz_point=size(read_volume); %it extracts the information about how many volumes were quantified

radius_nn(1:siz_point(1))=0; %it creates a vector that will contain the estimated radius of all the detected volumes

radius_nn=1000*( read_volume(:,1)*3/(4*pi)).^(1/3);

% the radius is calculated approximating the quantified volumes with be the volumes of a sphere

Mean_radius_nm = mean(radius_nn) % displays the mean radius of the segmented volumes

%please note that the radius of the segmented volumes is a good

%approximation of the full width at half maximum of the fluorescence object

Variance_radius_n = std(radius_nn) % displays the variance of the radius of the segmented volumes

%%%%%%%%%%%%%%%%%%%%%%%%%%%%%%%%%%%%%%%%%%%%%%%%%%%%%%%%%%%%%%%%%%%%%%%%%%%%%%%%%%%%%%%%%%%%%

%%%%%%%%%%%%%%%%%%%%%%%%%%%%%%%%%%%%%%%%%%%%%%%%%%%%%%%%% determination of

%%%%%%%%%%%%%%%%%%%%%%%%%%%%%%%%%%%%%%%%%%%%%%%%%%%%%%%%% the distance between the segmented volumes and sorting them between volumes belonging to the same cluster and volumes not belonging to the same cluster.

center_of_mass=xlsread(filename,5);% open page where the coordinate of the center of mass of the segmented volumes are listed

total_num=size(center_of_mass); % again number of detected spots just to check if it match with the previous values (not necessary)

total_resolved_spots = total_num(1) % displays the total number of volumes quantified

center_of_mass=xlsread(filename,5); % open the page of the file where the values of the centre of mass of the detected volumes are stored and write them into a matrix

distances(1:siz_point(1),1:siz_point(1))=0;% produce the matrix were the distances between detected volumes will be stored

cluster(1:siz_point(1),1:siz_point(1))=0;%produce a matrix where will be stored the information wether two segmented volumes are close together (distance fulfil cluster parameter) cluster (i,j)=1

%or not cluster(i,j)=0;

%%calculates the matrix of the distance between segmented volumes i and j as the distance between their centre of mass

%the factor 1000 converts the distance from micronmeter to nanometer

for i=1:siz_point(1)

for j=1:siz_point(1)

distances(i,j)= sqrt( (center_of_mass(i,1)-center_of_mass(j,1))^2 + (center_of_mass(i,2)-center_of_mass(j,2))^2 + (center_of_mass(i,3)-center_of_mass(j,3))^2 )*1000;

end

end

%Here is determined if two volumes are clustering together i.e. if their distance <= the sum of their radius multiplied for clust_par defined above, or not

for i=1:siz_point(1)

for j=1:siz_point(1)

if i==j

cluster(i,j)=0;

else

if distances(i,j)<=clast_par*(radius_nn(i)+radius_nn(j))

cluster(i,j)=1;

end

end

end

end

cluster3=cluster; %%%here the cluster matrix is doubled for keep avaliable its information at the end of the script

%%%%%%%%%%%%%%%%%%%%%%%%%%%%%%%%%%%%%%%%%%%%%%%%%%%%%%%%%%%%%%%%%%%%%%%%%%%%%%%%%%%%%%%%%%%%%%%%%%%%%%%%%%%%%%%%%%%

%%%%%%%%%%%%%%%%%%%%%%%%%%%%%%%%%%%%%%%%%%%%%%%%%%%%%%%%%%%%%%%%%%%%%%%%%%%%%%%%%%%%%%%%%%%%%%%%%%%%%%%%%%%%%%%%%%%%%%

% determination of the number of clusters the cluster size i.e. how many

% volumes are contained into one cluster and other cluster information

numclast=0;numclast2(1:siz_point(1),1)=1;numclast3(1:siz_point(1),1:siz_point(1))=0;

num=0; % initial number of clusters

cluster_record(1:siz_point(1),1:siz_point(1))=0;% matrix where the label of the cluster members should be stored i.e the label

%of the volumes of cluster members. Please note that the column number of cluster_record matrix is also the label of the first volume find for a given

%cluster if this column is not made only by zero values

% searching and isolating clusters

for i=1:siz_point(1)

count=0;% how many volumes are clustering with an individual volume

count3=0; %how many volumes are belonging to the same cluster

if sum( cluster(i,:))>=1

num=num+1;

% ind= sum( cluster(i,:));

for j=1:siz_point(1)

if cluster(i,j)==1

count=count+1;

count3=count3+1;

cluster(i,j)=0;% every time if a point on the cluster matrix is found to belong to a specific cluster it is set to

%zero in order to not choose this point twice. At the end of the cluster search the cluster matrix should be a full zeros matrix

cluster(j,i)=0;% everytime if a point on the cluster matrix is found to belong to a specific cluster is set to

%zero in order to the choose this point twice at the end the cluster matrix should be a full zeros matrix

vect(count)=j;% here are listed the label of the volumes clastering with the volume under analysis

cluster_record(count3,i)=j;

end

end

end

while count > 0;%%%% in this recursive loop are searched the volumes that were clustering with the first one and in case

%they are found clustering with other volumes these are stored and this cycle is repeated until when no more clustering volume are found

count2=count;

count=0;

vect2=vect;% saving the information contained in vect (is a variable size vector so overwriting is not to suggest)

clear vect % delete this variable in a way it can be defined newly in the following part of the loop

for ss=1:count2

for j=1:siz_point(1)

if cluster(vect2(ss),j)==1

count=count+1;

cluster(vect2(ss),j)=0;

cluster(j,vect2(ss))=0;

vect(count)=j;

ll=0; %%ll=0 a volume is for the first time found to be member of a given cluster; ll=1 the volume was already found to be part of the cluster

% and it will be not counted twice

for ms=1:count3

if cluster_record(ms,i)==j

ll=1;

end

end

if ll==0

count3=count3+1;

cluster_record(count3,i)=j;

end

ll=0;

end

end

end

clear vect2 % this variable vector will be redefined at the beginning of the loop

count;

end

if num > 0 % if a cluster is found

if count3>0

numb(num)=count3+1;% 1 comes from the fact that a cluster is always found starting from a volume that is not included

%in the counting routine

end

end

end

%%%%%%%%%%%%%%%%%%%%%%%%%%%%%%%%%%%%%%%%%%%%%%%%%%%%%%%%%%%%%%%%%%%%%%%%%%%%%%%%%%%%%%%%%%%%%%%%%

%%%%%%%%%%%%%%%%%%%%%%%%%%%%%%%%%%%%%%%%%%%%%%%%%%%%%%%%%%%%%%%%%%%%%%%%%%%%%%%%%%%%%%%%%%%%%%%

%%%%%%%%%%%%%%%%%%%%%%%%%%%%%%%dysplaying the information

sit=size(numb);

number_of_cluster=sit(2)% the number of columns of the variable numb gives the total number of clusters

total_number_of_spot_in_cluster= sum(numb)% the total number of volumes belonging to the one of the clusters (it must be inferior to the total number of volumes quantified!!!)

average_number_of_spot_in_cluster= mean(numb)% mean number of volumes belonging to a cluster

dispersion_number_of_spot_in_cluster= std(numb)% variance of the number of volumes belonging to a cluster

frequent_number_of_spot_in_cluster= median(numb)% median of the number of volumes belonging to a cluster

clust_info(1:sit(2),1:8)=0; % in this matrix some of the information is saved that will be exported later

%also the list of the cluster members and the coordinate of the centre of

%mass of the entire cluster

indd=0;

for l=1:siz_point(1)

cluster_member(1)=0;

if sum(cluster_record(:,l))>=1

indd=indd+1;

sol=1;

cluster_member(1)=l;% Variable vector that will contain all the label of the volumes the belonging to a given cluster

for m=1:1:siz_point(1)

if cluster_record(m,l)>=1

sol=sol+1;

cluster_member(sol)=cluster_record(m,l);

end

end

end

if cluster_member(1)>0

got=sol;% number of volumes in a given cluster

rel=0;

xx=0;

yy=0;

zz=0;

for uu=1:got% summing up x y z and z position of the cluster in nm (factor 1000)

xx=xx+1000*center_of_mass(cluster_member(uu),1);

yy=yy+1000*center_of_mass(cluster_member(uu),2);

zz=zz+1000*center_of_mass(cluster_member(uu),3);

end

% here is calculated the distance between two volumes in the same

% cluster. This distance is calculated twice volume i versus volume

% j and volume j versus volume i thus later a factor 0.5 will be

% applied to obtain the mean distance

for i=1:got

for j=1:got

dist(i,j)=sqrt( (center_of_mass(cluster_member(i),1)-center_of_mass(cluster_member(j),1))^2 + (center_of_mass(cluster_member(i),2)-center_of_mass(cluster_member(j),2))^2 + (center_of_mass(cluster_member(i),3)-center_of_mass(cluster_member(j),3))^2 )*1000;

if dist(i,j)>0

rel=rel+1;

disti(rel)=dist(i,j);% converts the matrix of distances into a vector

end

end

end

%%%% here the information that will be exported

clust_info(indd,1)=numb(indd) ; % number of volumes in each cluster

clust_info(indd,2)=0.5*mean(disti); % mean distances between volumes in the same cluster

clust_info(indd,3)=std(disti/2);% qualitative estimate of variance of distances between volumes in the same cluster

clust_info(indd,4)=max(disti);% max value of distance between volumes in the same cluster

clust_info(indd,5)=min(disti);% min value of distance between volumes in the same cluster

clust_info(indd,6)=xx/sol;% x coordinate of the cluster centre of mass

clust_info(indd,7)=yy/sol;% y coordinate of the cluster centre of mass

clust_info(indd,8)=zz/sol;% z coordinate of the cluster centre of mass

clear cluster_member sol

clear dist disti xx yy zz

end

end

%%% the labels of cluster members are saved in a two column matrix the

%%% second column contains the label of the first member of the cluster and

%%% it repeats its value until when the same cluster is under consideration.

%%% The first column displays the label of the other volumes belonging to

%%% the clusters

a=0;

for i=1:siz_point(1)

if sum(cluster_record(:,i))>=1

for j=1:siz_point(1)

if cluster_record(j,i)>=1

a=a+1;

position(a,1)=cluster_record(j,i);

position(a,2)=i;

end

end

end

end

position2= position-1; % since the labelling in Imaris starts from number 0 and not from one in order to find the same cluster in Imaris to the

% to the label values must be substructed 1

%

%%%%%%%% printing in the output file the data quantified till now

%%%%%%

names={'number of particles', 'mean distance', 'variance of distance', 'max distance', 'min distance', 'center_x', 'center_y', 'center_z' };

names2={'Mean_radius_nm', 'Variance_radius_n ', 'Total_resolved_spots' };

values=[Mean_radius_nm , Variance_radius_n total_resolved_spots];

xlswrite([num2str(clast_par) '_output_' filename '.xlsx'],names2,1,'A1')

xlswrite([num2str(clast_par) '_output_' filename '.xlsx'],values,1,'A2')

xlswrite([num2str(clast_par) '_output_' filename '.xlsx'],clust_info,2,'A2')

xlswrite([num2str(clast_par) '_output_' filename '.xlsx'],names,2,'A1')

xlswrite([num2str(clast_par) '_output_' filename '.xlsx'],position2,3,'A1')

%%%% quantification of the distance between different clusters

%%%%% the distance between clusters is defined to be the distance between their centre of

%%%%% mass

dist_clust(1:sit(2),1:sit(2))=0;

cc1=0;

for i=1:sit(2)

for j=1:sit(2)

if i==j

dist_clust(i,j)=100000;% the fact that the distance of one cluster respect itself is zero would make more complicate

%to determine the minimum distance between a cluster and it neighbours

else

cc1=cc1+1;

dist_clust(i,j)=sqrt( (clust_info(i,6)-clust_info(j,6))^2 + (clust_info(i,7)-clust_info(j,7))^2 + (clust_info(i,8)-clust_info(j,8))^2 );

dist_clust2(cc1)=dist_clust(i,j);

end

end

end

mindist_cluster=min(dist_clust);% the minimum distance between one cluster and the other ones

mean_dist_cluster=mean(mindist_cluster);% the mean distance between closer clusters

%%% exporting the latest information

xlswrite([num2str(clast_par) '_output_' filename '.xlsx'], dist_clust,4,'A1')

xlswrite([num2str(clast_par) '_output_' filename '.xlsx'],mean_dist_cluster,5,'A1')

xlswrite([num2str(clast_par) '_output_' filename '.xlsx'],mindist_cluster',5,'B1')

names3={'number_of_cluster', 'total_number_of_spot_in_cluster ', 'average_number_of_spot_in_cluster', 'dispersion_number_of_spot_in_cluster','frequent_number_of_spot_in_cluster' }

values3=[number_of_cluster total_number_of_spot_in_cluster average_number_of_spot_in_cluster dispersion_number_of_spot_in_cluster frequent_number_of_spot_in_cluster];

values3=values3';

xlswrite([num2str(clast_par) '_output_' filename '.xlsx'],names3,6,'A1')

xlswrite([num2str(clast_par) '_output_' filename '.xlsx'],values3',6,'A2')
